# Combined genetic analysis of juvenile idiopathic arthritis clinical subtypes identifies novel risk loci, target genes and key regulatory mechanisms

**DOI:** 10.1136/annrheumdis-2020-218481

**Published:** 2020-10-26

**Authors:** Elena López-Isac, Samantha L Smith, Miranda C Marion, Abigail Wood, Marc Sudman, Annie Yarwood, Chenfu Shi, Vasanthi Priyadarshini Gaddi, Paul Martin, Sampath Prahalad, Stephen Eyre, Gisela Orozco, Andrew P Morris, Carl D Langefeld, Susan D Thompson, Wendy Thomson, John Bowes

**Affiliations:** 1 Centre for Genetics and Genomics Versus Arthritis, Centre for Musculoskeletal Research, Manchester Academic Health Science Centre, The University of Manchester, Manchester, UK; 2 Center for Public Health Genomics and Department of Biostatistical Sciences, Wake Forest University School of Medicine, Winston-Salem, North Carolina, USA; 3 Division of Rheumatology, Cincinnati Children's Hospital Medical Center and University of Cincinnati, Cincinnati, Ohio, USA; 4 The Lydia Becker Institute of Immunology and Inflammation, Faculty of Biology, Medicine and Health, The University of Manchester, Manchester, Manchester, UK; 5 Department of Pediatrics and Human Genetics, Emory University, and Children’s Healthcare of Atlanta, Atlanta, Georgia, USA; 6 National Institute of Health Research Manchester Biomedical Research Centre, Manchester Academic Health Science Centre, Manchester University NHS Foundation Trust, Manchester, Greater Manchester, UK

**Keywords:** arthritis, juvenile, polymorphism, genetic, biological therapy

## Abstract

**Objectives:**

Juvenile idiopathic arthritis (JIA) is the most prevalent form of juvenile rheumatic disease. Our understanding of the genetic risk factors for this disease is limited due to low disease prevalence and extensive clinical heterogeneity. The objective of this research is to identify novel JIA susceptibility variants and link these variants to target genes, which is essential to facilitate the translation of genetic discoveries to clinical benefit.

**Methods:**

We performed a genome-wide association study (GWAS) in 3305 patients and 9196 healthy controls, and used a Bayesian model selection approach to systematically investigate specificity and sharing of associated loci across JIA clinical subtypes. Suggestive signals were followed-up for meta-analysis with a previous GWAS (2751 cases/15 886 controls). We tested for enrichment of association signals in a broad range of functional annotations, and integrated statistical fine-mapping and experimental data to identify target genes.

**Results:**

Our analysis provides evidence to support joint analysis of all JIA subtypes with the identification of five novel significant loci. Fine-mapping nominated causal single nucleotide polymorphisms with posterior inclusion probabilities ≥50% in five JIA loci. Enrichment analysis identified RELA and EBF1 as key transcription factors contributing to disease risk. Our integrative approach provided compelling evidence to prioritise target genes at six loci, highlighting mechanistic insights for the disease biology and *IL6ST* as a potential drug target.

**Conclusions:**

In a large JIA GWAS, we identify five novel risk loci and describe potential function of JIA association signals that will be informative for future experimental works and therapeutic strategies.

**Key messages**

**What is already known about this subject?**

- Juvenile idiopathic arthritis (JIA) is the most common form of childhood arthritis. However, our understanding of the genetic basis of JIA is hampered by low disease prevalence and extensive clinical heterogeneity represented by seven disease subtypes, with only 17 known susceptibility loci to date.

**What does this study add?**

- Although JIA is a heterogeneous disease, we show that most susceptibility loci are shared across multiple clinical subtypes, enabling joint analysis of clinically related subtypes, both for this study and future projects, increasing the power of our study leading to the identification of five novel susceptibility loci in the largest genome-wide genetic study to date.
- By linking susceptibility genetic variants to target genes, integrating functional annotations, statistical fine mapping, expression data from 15 immunological cell types and chromatin interaction data (HiChIP and Hi-C) from human T and B cell types, we identify putative causal (i) single nucleotide polymorphisms; (ii) genes and (iii) cell types while also highlighting key regulatory mechanisms underlying disease.

**Key messages**

**How might this impact on clinical practice or future developments?**

- The results of this study demonstrate that clinically heterogeneous subtypes can be analysed in a combined approach to identify novel shared susceptibility loci which is an approach that will be informative for genetic studies of other clinically heterogeneous diseases.
- We identify causal genes at JIA susceptibility loci which is an essential step in the translational of genetic discoveries to clinical benefit by highlighting potential therapeutic targets.

## Introduction

The contribution of large-scale genetic studies to the understanding of pathogenesis and management of complex traits has been widely documented over the last decade with the identification of thousands of genetic associations and their subsequent implications for biological pathways, drug discovery and repurposing.^1 2^ However, progress in low-prevalence diseases has not been as rapid owing to hindrances in the recruitment of well-powered cohorts. This is well illustrated by considering the distinct and heterogeneous forms of childhood arthritis that are clinically encompassed under the term of juvenile idiopathic arthritis (JIA). JIA comprises childhood rheumatic conditions characterised by inflammatory arthritis of unknown origin that persists for at least 6 weeks and begins before the age of 16 years.^3^ The International League of Associations for Rheumatology (ILAR) distinguishes seven JIA subtypes: oligoarticular arthritis (oligoJIA); rheumatoid factor (RF)-negative polyarthritis (RF–polyJIA); RF-positive polyarthritis (RF+polyJIA); juvenile psoriatic arthritis (JPsA); enthesitis-related arthritis childhood spondyloarthropathy (ERA); systemic arthritis (sJIA); and undifferentiated arthritis.^4^

To date genetic studies in JIA susceptibility have identified 17 genome-wide significant associations highlighting a number of key findings^5–7^ including, first, that there is overlap of susceptibility loci between two of the most common JIA subtypes, oligoJIA and RF–polyJIA; specifically, in the human leucocyte antigen (HLA) region, these two subtypes share the presence of a glycine at amino acid position 13 of HLA-DRB1 as their highest risk factor, resembling the findings in adult seronegative rheumatoid arthritis (RA).^6 7^ Together these two JIA subtypes define a genetically homogeneous cluster sometimes referred to with the term ‘polygo’.^7–9^ Second, this high genetic correlation is not as evident in the remaining subtypes, especially considering their divergent associations observed across the HLA region. For example, the presence of a histidine at the same HLA-DRB1 position confers the highest risk for RF+polyJIA, consistent with the association reported in adult seropositive RA.^7^ In addition, the amino acid at position 58 of HLA-DRB1 has been shown to be a specific risk factor for sJIA.^10^

The clinical heterogeneity of JIA remains a challenging issue in deciphering its genetic architecture by balancing the need to focus on more clinically/genetically homogeneous subtypes against potentially sacrificing sample size. As a result, there has been a tendency to address the genetics of JIA in a subtype-based manner.^6–9^ However, multinomial approaches have recently been developed to overcome the heterogeneity problem by allowing exploration of the genetic relationships between multi-phenotype categories.^11^ In this study, we hypothesised that a genome-wide association study (GWAS) combining all JIA subtypes would optimise the success rate in locus discovery. We, therefore, performed a new genome-wide scan of ~7.5 million single nucleotide polymorphisms (SNPs) in the largest JIA GWAS cohort recruited to date, and implemented a novel approach to systematically investigate specificity and sharing of associated loci across ILAR subtypes to support our strategy.

## Methods

### Study cohort and GWAS quality control

A total of 4520 UK JIA samples and 9965 healthy individuals were recruited for the present study. JIA DNA samples were genotyped on the Illumina Infinium CoreExome and Infinium OmniExpress genotyping arrays. Sample-level quality control (QC) was applied based on the following exclusion criteria: call rate <0.98 and discrepancy between genetically inferred sex and database records. SNPs that were non-autosomal, had a call rate <0.98 or a minor allele frequency (MAF) <0.01 were excluded. Healthy controls were genotyped using the Illumina Infinium CoreExome genotyping array. QC was consistent with that described above for JIA samples.

Identity-by-descent was used to identify related individuals across all study samples. For each related pair, the sample with the highest call rate was retained. Outliers were identified and excluded based on ancestry using principal component (PC) analysis performed with the flashpca software package (V.2.0) where outliers were identified using aberrant R library (V.1.0).^12 13^

The total number of individuals that remained in the final QC-filtered data set was 12 501 (3305 cases and 9196 healthy controls) (online supplemental table 1).

10.1136/annrheumdis-2020-218481.supp1 Supplementary data

### Imputation

The QC-filtered GWAS data set was subjected to whole-genome genotype imputation. Haplotype phasing and imputation were performed in the Michigan Imputation server using SHAPEIT2^14^ and Minimac3,^15^ respectively, and the Haplotype Reference Consortium reference panel. Following imputation, SNPs were excluded based on MAF <0.01 and imputation quality (r^2^) <0.4.

### Association testing and meta-analysis

Case-control association testing was performed by SNPTEST software package (V.2.5.2). Three PCs were included as covariates to account for any residual population substructure. Any SNP with a p value <5 x 10^-6^ was selected for validation in GWAS summary statistics from an independent data set of 2751 JIA cases (oligoJIA and RF–polyJIA) and 15 886 controls of European ancestry.^8^ An inverse variance weighted fixed effects meta-analysis was performed using the software package GWAMA (V.2.2.2).^16^ The presence of heterogeneity of ORs across data sets was evaluated with the test statistics I^2^ and Q.

### Clinical subtype specificity

The specificity and sharing of JIA susceptibility SNPs across ILAR subtypes was interrogated using Bayesian multinomial logistic regression assuming an additive model implemented in the software package Trinculo (V.0.96).^11^ Model selection for specificity or sharing was based on comparison of log-Bayes factors (logBFs) where a positive logBF was interpreted as evidence that a particular association is specific to an ILAR subtypes, and vice versa.

### Statistical fine-mapping of JIA-associated loci

Statistical fine-mapping of the association signal within each locus was performed using the FINEMAP software package (V.1.3.1).^17^ The method estimates the posterior inclusion probabilities (PIPs) for SNPs to be causal, which in turn were used to generate 95% credible SNP sets for each locus (the smallest list of variants that jointly have a probability of including the causal variant ≥95%).

### Functional annotation enrichment analysis

Summary statistics from the GWAS including all ILAR subtypes were tested for enrichment in four categories of annotations based on experimental genomic data including gene structure (coding sequence (CDS), 3‘UTR and 5‘UTR) from the GENCODE Project, binding sites for 165 transcription factors (TFs) from the ENCODE Project, and enhancers and active promoters for 98 cell types derived from the Roadmap Epigenomics Project.^18–20^ Enrichment of JIA associations were tested separately in each annotation using fgwas (V.0.3.6).^21^ A joint model of independent enrichments was further identified using the cross-validation likelihood option implemented in fgwas.

### Gene prioritisation

Expression quantitative trait locus (eQTL) data for 15 immune cell types was downloaded from the DICE (Database of Immune Cell Expression, eQTLs and Epigenomics) project website.^22^ Correlation of susceptibility association signals and gene expression were identified by selecting the top eQTL SNP for each gene and retaining those that were also present in the combined list of all credible SNPs. This analysis was further supported by statistical colocalisation of association and eQTL signals. The identification of the target genes of JIA-associated regions was further complemented by the interrogation of high-resolution maps of chromatin interactions for SNPs correlated with eQTL signals using H3K27ac HiChIP data in B and T cells.^23^ We also explored chromatin interaction maps obtained by capture Hi-C.^24^

Additional details of the Methods are available in the online supplemental material.

10.1136/annrheumdis-2020-218481.supp2 Supplementary data

## Results

### Five novel susceptibility loci for JIA

**Figure 1:**
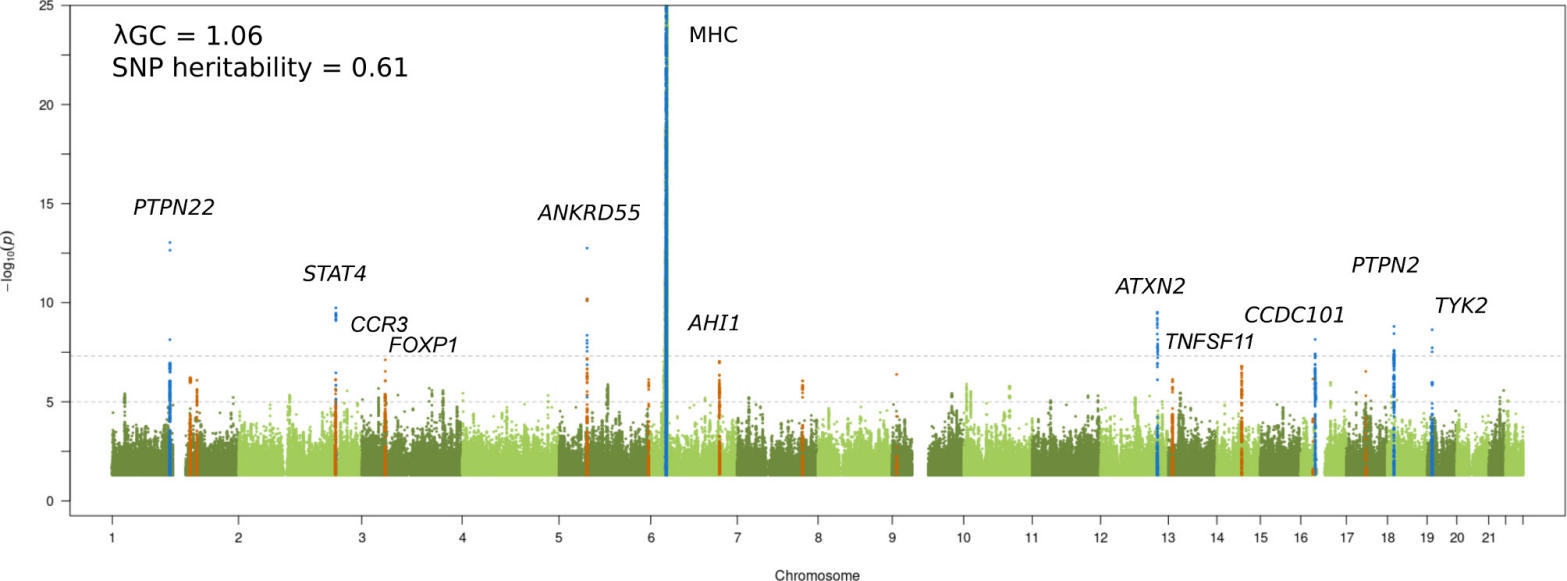
Manhattan plot representing the JIA GWAS results. The −log_10_ of the p values are plotted against their physical chromosomal position. The upper and lower lines represent the genome-wide significance level (p≤5 × 10^−8^) and p value threshold at p≤1 × 10^−6^, respectively. The plot has been truncated at p≤1 × 10^−25^. Genome-wide significant associations are coloured blue and suggestive significance are coloured orange. Genome-wide significant loci and the suggestive signals that reached p≤5 × 10^−8^ after the replication step are labelled. The genomic inflation factor (λ_GC_) estimated on the complete data set was 1.06, with a rescaled λ_1000_ of 1.01 indicating minimal residual population stratification based on inflation of test statistics. The SNP-based heritability for JIA susceptibility was estimated to be 0.61 (SE 0.04). JIA, juvenile idiopathic arthritis; GWAS, genome-wide association study; SNP, single nucleotide polymorphism.

We performed a JIA GWAS comprising 12 501 individuals (3305 cases and 9196 healthy controls) and a high-density SNP panel with 7 461 861 variants. The combined analysis of all available JIA cases identified eight loci reaching genome-wide significance (p≤5 x 10^-8^), of which seven have previously been reported and recognised by the notable gene at each locus: MHC (6p25-p34), *PTPN22* (1p13.2), *STAT4* (2q32.2-q32.3), *ANKRD55* (5q11.2), *ATXN2* (12q24.12), *PTPN2* (18p11.21), and *TYK2* (19p13.2) (figure 1 and table 1).^6^ The strongest association was found to SNPs within the extended MHC region (chr6: 28 477 797 to 33 448 354). An in-depth analysis of this region has previously been reported for JIA, including a subset of samples from the current study; therefore this study will focus on non-MHC associations.^7^ The novel genome-wide significant association was represented by the lead SNP rs497523 (p=7.12 x 10^-9^), which is intronic to *CCDC101* (16p11.2), also known as *SGF29*. A further 37 lead SNPs from independent loci, based on linkage disequilibrium, reached the suggestive significance threshold (p≤5 x 10^-6^). This included previously reported and potentially novel JIA loci (table 1). Summary statistics for 22 of these variants were available from a previously published JIA GWAS comprising 2751 cases and 15 886 controls^8^ and meta-analysed with the current GWAS data. The meta-analysis identified a further four novel SNPs exceeding genome-wide significance in the proximity of the genes *AHI1* (6q23.3), *CCR3* (3p21.31), *TNFSF11* (13q14.11) and *FOXP1* (3p13) (table 2 and online supplemental table 2). Hence, a total of five new signals associated with JIA were identified. In addition, three SNPs showed evidence for replication in the independent data set although the meta-analysis test statistic did not exceed genome-wide significance (table 2): *TNFSF8* (rs7043505) (*_meta_*p=8.27 x 10^-8^), *AFF3* (rs11692867) (*_meta_*p=9.26 x 10^-8^), and *RUNX3* (rs72657048) (*_meta_*p=3.51 x 10^-7^). There was no evidence of between-study heterogeneity at any of these loci.

**Table 1:** Non-HLA index SNPs passing study suggestive significance threshold (5×10^-6^) and genome-wide significance threshold

SNP	Chr.	Position (bp)	Notable genes	Risk/non-risk allele	RAF	HWE (cases)	HWE (controls)	P value	OR	95% CI
**rs6679677**	**1**	114 303 808	***RSBN1; PTPN22***	**A/C**	**0.1**	**0.19**	**0.35**	**9.18E-14**	**1.36**	**1.24 to 1.48**
**rs7731626**	**5**	55 444 683	***ANKRD55***	**G/A**	**0.63**	**0.27**	**0.38**	**1.76E-13**	**1.22**	**1.15 to 1.3**
**rs11889341**	**2**	191 943 742	***STAT4***	**T/C**	**0.22**	**0.96**	**1**	**1.83E-10**	**1.24**	**1.16 to 1.32**
**rs4766578**	**12**	111 904 371	***ATXN2***	**T/A**	**0.49**	**0.21**	**0.13**	**3.03E-10**	**1.22**	**1.15 to 1.29**
**rs9960807**	**18**	12 770 851	***RP11-973H7.1;PTPN2***	**G/A**	**0.13**	**0.74**	**0.46**	**1.58E-09**	**1.26**	**1.16 to 1.36**
**rs34536443**	**19**	10 463 118	***TYK2***	**G/C**	**0.95**	**0.77**	**0.56**	**2.32E-09**	**1.53**	**1.31 to 1.79**
**rs497523**	**16**	28 577 931	***CLN3; CCDC101***	**T/C**	**0.65**	**0.94**	**0.03**	**7.12E-09**	**1.17**	**1.11 to 1.25**
rs13160933	5	55 545 859	*NA*	C/T	0.88	0.84	1	6.49E-08	1.26	1.15 to 1.38
rs79815064	3	46 277 577	*CCR3*	A/G	0.87	0.45	0.6	7.61E-08	1.25	1.14 to 1.37
rs2614258	6	135 677 202	*AHI1*	A/G	0.38	0.2	0.22	9.17E-08	1.15	1.08 to 1.22
rs1051533	14	69 259 662	*ZFP36L1*	A/C	0.21	0.63	0.85	1.62E-07	1.2	1.12 to 1.28
rs113171555	17	38 296 272	*CASC3*	A/G	0.02	0.73	0.14	2.92E-07	1.46	1.22 to 1.75
rs72704368	9	8 894 396	*PTPRD*	A/G	0.05	0.76	0.64	4.14E-07	1.3	1.15 to 1.47
rs2481065	1	154 311 911	*ATP8B2; IL6R*	G/A	0.11	0.32	0.87	6.11E-07	1.24	1.14 to 1.35
rs77011494	16	24 333 566	*CACNG3*	A/G	0.04	0.86	0.89	7.04E-07	1.41	1.23 to 1.6
rs7320806	13	27 684 929	*USP12*	C/A	0.09	0.86	0.12	7.36E-07	1.25	1.14 to 1.37
rs6434390	2	191 262 762	*INPP1; MFSD6*	G/C	0.48	0.05	0.54	7.42E-07	1.16	1.1 to 1.23
rs12654812	5	176 794 191	*RGS14*	A/G	0.34	0.22	0.64	7.61E-07	1.17	1.1 to 1.24
rs840012	1	167 414 872	*CD247*	C/T	0.59	0.46	0.95	8.21E-07	1.15	1.08 to 1.22
rs12706860	7	128 570 026	*NA*	C/G	0.65	0.6	0.13	8.78E-07	1.18	1.11 to 1.25
rs7204355	16	58 951 694	*RP11-410D17.2*	G/T	0.79	0.26	0.07	1.04E-06	1.19	1.1 to 1.28
rs706778	10	6 098 949	*IL2RA*	T/C	0.4	0.34	0.78	1.28E-06	1.15	1.09 to 1.22
rs4869314	5	96 229 225	*ERAP2*	G/T	0.49	0.58	0.88	1.35E-06	1.14	1.08 to 1.21
rs7082720	10	90 742 049	*ACTA2*	T/C	0.45	0.6	0.66	1.67E-06	1.15	1.09 to 1.21
rs2222138	18	12 889 217	*PTPN2*	G/T	0.68	0.42	0.98	2.02E-06	1.17	1.1 to 1.24
rs1521088	3	132 815 094	*TMEM108*	T/C	0.02	0.74	0.06	2.08E-06	1.41	1.18 to 1.68
rs34173901	3	33 087 914	*GLB1*	C/G	0.15	0.3	0.65	2.13E-06	1.2	1.12 to 1.3
rs76870128	3	138 211 845	*CEP70*	C/T	0.97	0.57	0.53	2.66E-06	1.61	1.31 to 2
rs58923164	21	44 158 451	*PDE9A*	T/G	0.04	1	1	2.68E-06	1.34	1.17 to 1.53
rs13433914	3	159 902 148	*IL12A-AS1*	C/G	0.22	0.71	0.63	2.74E-06	1.17	1.09 to 1.25
rs2371887	2	214 085 179	*NA*	G/A	0.43	0.33	0.97	2.79E-06	1.15	1.08 to 1.21
rs1717501	10	14 354 673	*FRMD4A*	C/A	0.12	0.57	0.55	3.07E-06	1.23	1.13 to 1.34
rs138815617	17	19 445 425	*SLC47A1*	A/G	0.01	0.6	1	3.28E-06	1.54	1.22 to 1.94
rs12430303	13	43 032 027	*TNFSF11*	C/T	0.45	0.4	0.57	3.61E-06	1.13	1.07 to 1.2
rs186715000	4	1 589 324	*NA*	G/A	0.01	1	0.27	3.72E-06	1.52	1.24 to 1.87
rs7043505	9	117 628 528	*TNFSF8*	A/G	0.55	0.47	0.25	3.74E-06	1.15	1.08 to 1.21
rs72657048	1	25 289 734	*RUNX3*	G/C	0.5	0.65	0.77	3.90E-06	1.14	1.08 to 1.21
rs7647909	3	71 200 157	*FOXP1*	G/T	0.24	0.13	0.6	4.56E-06	1.16	1.09 to 1.23
rs11692867	2	100 759 477	*AFF3*	G/A	0.64	0.53	0.6	4.57E-06	1.13	1.07 to 1.2
rs80136777	3	45 931 005	*CCR9*	T/A	0.88	0.57	0.2	4.68E-06	1.2	1.1 to 1.32
rs139529714	4	169 369 671	*DDX60L*	C/T	0.01	1	0.27	4.78E-06	1.52	1.24 to 1.87
rs521786	11	129 607 371	*NA*	C/A	0.11	0.65	0.11	4.94E-06	1.19	1.09 to 1.3
rs661171	11	110 016 519	*ZC3H12C*	G/T	0.72	0.82	0	4.95E-06	1.16	1.09 to 1.24
rs6506561	18	8 233 559	*PTPRM*	T/C	0.55	0.86	0.1	5.00E-06	1.13	1.07 to 1.19

Genome-wide significant loci for juvenile idiopathic arthritis are highlighted in bold.

bp, base pair; Chr., chromosome; HLA, human leucocyte antigen; HWE, Hardy-Weinberg equilibrium; RAF, risk allele frequency; SNP, single nucleotide polymorphism.

**Table 2:** SNP showing genome-wide significant or suggestive associations with juvenile idiopathic arthritis in the meta-analysis

SNP	Chr.	Position (bp)	Notable genes	Risk/non-risk allele	UK GWAS P value	USA GWAS P value	META P value	META OR	META 95% CI	Q statistic	Q P value	I2
rs2614258	6	135 677 202	*AHI1*	A/G	9.17E-08	6.50E-06	9.47E-12	1.17	1.12–1.22	0.18	0.67	0
rs79815064	3	46 277 577	*CCR3*	A/G	7.61E-08	8.43E-05	3.31E-11	1.25	1.17–1.34	0.87	0.35	0
rs12430303	13	43 032 027	*TNFSF11*	C/T	3.61E-06	9.23E-04	1.88E-09	1.14	1.09–1.19	0.56	0.45	0
rs7647909	3	71 200 157	*FOXP1*	G/T	4.56E-06	5.27E-05	2.02E-09	1.17	1.11–1.23	0	0.96	0
rs7043505	9	117 628 528	*TNFSF8*	A/G	3.74E-06	0.008333	8.27E-08	1.12	1.07–1.17	1.09	0.3	0.08
rs11692867	2	100 759 477	*AFF3*	G/A	4.57E-06	0.004152	9.26E-08	1.13	1.08–1.19	0.59	0.44	0
rs72657048	1	25 289 734	*RUNX3*	G/C	3.90E-06	0.008138	3.51E-07	1.13	1.08–1.18	0.69	0.41	0

bp, base pair; Chr., chromosome; GWAS, genome-wide association study; SNP, single nucleotide polymorphism.

### Evidence for shared non-HLA loci across JIA clinical subtypes

We systematically addressed the genetic relationship across ILAR subtypes in a Bayesian framework. We performed a Bayesian model selection between the best subgroup-specific model and the best sharing model and estimated the logBFs for specificity of effects at each locus. The analysis included the 44 non-HLA index SNPs passing study suggestive significance threshold (5×10^-6^) (table 1). The results revealed evidence for sharing of JIA susceptibility loci across multiple ILAR subtypes since most of the analysed SNPs showed negative logBFs for specificity. This pattern was also evident for previously reported JIA susceptibility SNPs based on a combined cohort of oligoJIA and RF-polyJIA subtypes (online supplemental table 3, online supplemental figure 1). Moreover, the vast majority of the strongest logBFs (values between −4.5 and −9) were observed for the sharing model that comprised all JIA subtypes. Only seven loci (16%) showed weak evidence in favour of them being specific to the polygo subgroup (logBF of 0.06 to 0.5). Overall, these findings support our approach of performing a joint analysis of all available JIA cases to maximise power to detect novel susceptibility loci.

### Enrichment of JIA susceptibility SNPs in TFBS and cell-type specific regulatory regions

**Figure 2:**
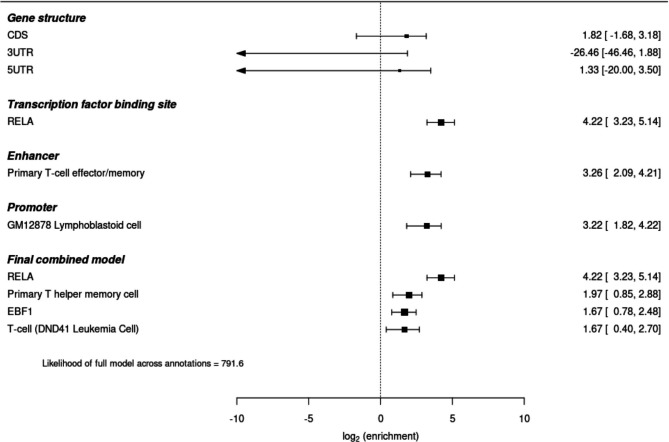
Functional enrichment analysis for JIA associations. Forest plot representing enrichment analysis results across four annotation categories based on experimental functional genomic data, and the final statistical annotation model. JIA, juvenile idiopathic arthritis.

We investigated the over-representation of JIA susceptibility SNPs in functional categories including gene structure (CDS, 3‘UTR and 5‘UTR), transcription factor binding sites (TFBSs) and enhancers and active promoters in 98 cell/tissue types. Our results showed no evidence for significant enrichment of JIA susceptibility SNPs in any of the gene structure annotations (p values>0.1) (figure 2). The most significant enrichment was found to binding sites for the TF RELA (p value=2.66 x 10^-8^) (online supplemental table 4 and online supplemental figure 2). Additionally 52 out of the 165 TFBSs interrogated showed significant over-representation, including EBF1 (p value=6.00 x 10^-6^), BATF (p value=1.51 x 10^-4^) and FOXA2 (p value=9.06 x 10^-4^). Enrichment of JIA susceptibility SNPs was also identified to cell-type specific enhancers in three broad tissue types: blood, thymus, and gastrointestinal tract (online supplemental table 5, online supplemental figure 3). Specially, JIA SNPs showed over-representation of enhancers in different subsets of T cells, pointing to primary effector/memory T cells, primary T helper memory cells, primary T helper 17, primary natural killer and primary T regulatory cells as key players for JIA pathogenesis. Enrichment in active promoters was observed in a wider range of tissue/cell types, with GM12878 lymphoblastoid B cells showing the strongest over-representation (p value=1.06 x 10^-3^) (online supplemental table 6, online supplemental figure 4).

**Figure 3:**
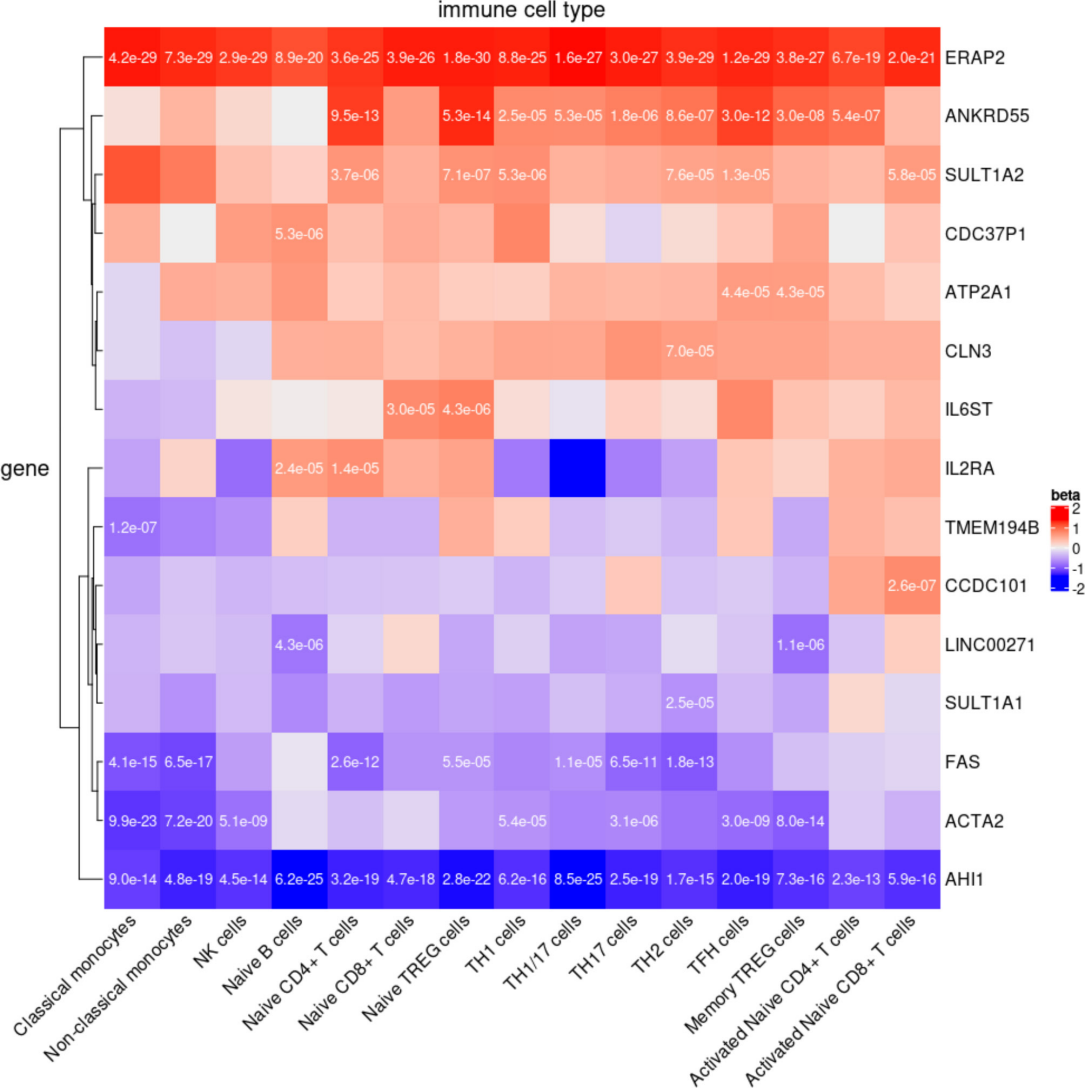
eQTL analysis. Significant eQTL from 15 disease relevant cell types of the DICE database including three innate immune cell types (classical monocytes, non-classical monocytes and natural killer cells), four adaptive immune cell types that have not encountered cognate antigen in the periphery (naive B cells, naive CD4^+^ T cells, naive CD8^+^ T cells and naive regulatory T cells (Treg)), six CD4^+^ memory or more differentiated T cell subsets (Th1, Th1/17, Th17, Th2, follicular helper T cell (Tfh) and memory Treg), and two activated cell types (naive CD4^+^ and CD8^+^ T cells that were stimulated ex vivo). The p value for significant correlations are reported in each cell for credible SNPs that capture the most significant eQTL. Beta coefficients to illustrate direction and magnitude as determined by risk allele. eQTL, expression quantitative trait locus; SNPs, single nucleotide polymorphisms.

Given the expected correlation between the analysed annotations, we then proceeded to perform a stepwise selection process to select a subset of non-redundant annotations. A combined model derived from all categories of annotations consisted of binding sites for RELA and EBF1, and enhancers in primary T helper memory cells and the T cell leukaemia cell line DND-41. The maximum likelihood of this cross-category model exceeded that from any of the single-annotation models thus identifying the most statistically relevant regulatory elements (figure 2). No annotations in the final model were excluded with cross-validation.

### Prioritising potential causal SNPs

Using our high-density SNP panel, we aimed to identify the putative causal SNPs driving the association signals. For this purpose, we applied a Bayesian fine-mapping approach^17^ to define the PIP of each variant being causal given all other variants in the region. We fine-mapped each of the five newly discovered loci and 12 previously reported non-MHC susceptibility loci (p value<5 x 10^-6^ in the present study) to identify 95% credible SNP sets. There was no evidence to support multiple distinct association signals at any locus. For 5 (29%) and 10 (59%) of the 17 loci, fine-mapping resolved the association signal to 95% credible sets of ≤10 and ≤30 causal variants, respectively (online supplemental tables 7 and 8). Moreover, we identified five SNPs with PIPs of at least 0.5 for the following loci: *RSBN1-PTPN22* (1p13.2; rs6679677), *FOXP1* (3p13; rs7647909), *CCR3* (3p21.31; rs79815064), *ANKRD55* (5q11.2; rs7731626) and *TYK2* (19p13.2; rs34536443) (online supplemental table 7). Interestingly, the method was able to identify rs34536443, a well-characterised non-synonymous variant in autoimmunity,^25^ as the likely causal variant for *TYK2* locus with a PIP of 80%.

### Prioritising target genes

The identification of the target genes of the disease-associated variants is a crucial step towards describing the biological impact of a statistical association. To address this question, we first used eQTL data derived from 15 disease relevant immune cell types to correlate the identified credible SNPs with genes in each locus. The credible SNP sets captured the lead eQTL SNP for 15 genes (eGenes) at nine loci (figure 3 and online supplemental table 9). These observations were supported by statistical colocalisation (online supplemental table 10). Subsequently, we complemented the identification of the putative target genes of JIA SNPs by analysing high-resolution maps of enhancer-promoter interactions in human B and T cells. We observed HiChIP interactions for the promoters of 6 out of the 15 JIA eGenes: *IL2RA*, *CLN3*, *ATP2A1*, *IL6ST*, *CCDC101* (*SGF29*) and *ERAP2* (online supplemental table 11). In addition, *SULT1A2*, *SULT1A1*, *ACTA2*, *FAS* and *AHI1* promoters were located within 1 kb windows of JIA credible SNPs that overlapped an H3K27ac peak as identified from HiChIP data. We also observed promoter interactions for JIA credible SNPs and the promoters of *IL2RA*, *CLN3*, *IL6ST*, *CCDC101* and *ERAP2* through chromatin interaction maps obtained by capture Hi-C experiments (online supplemental table 12).

**Figure 4:**
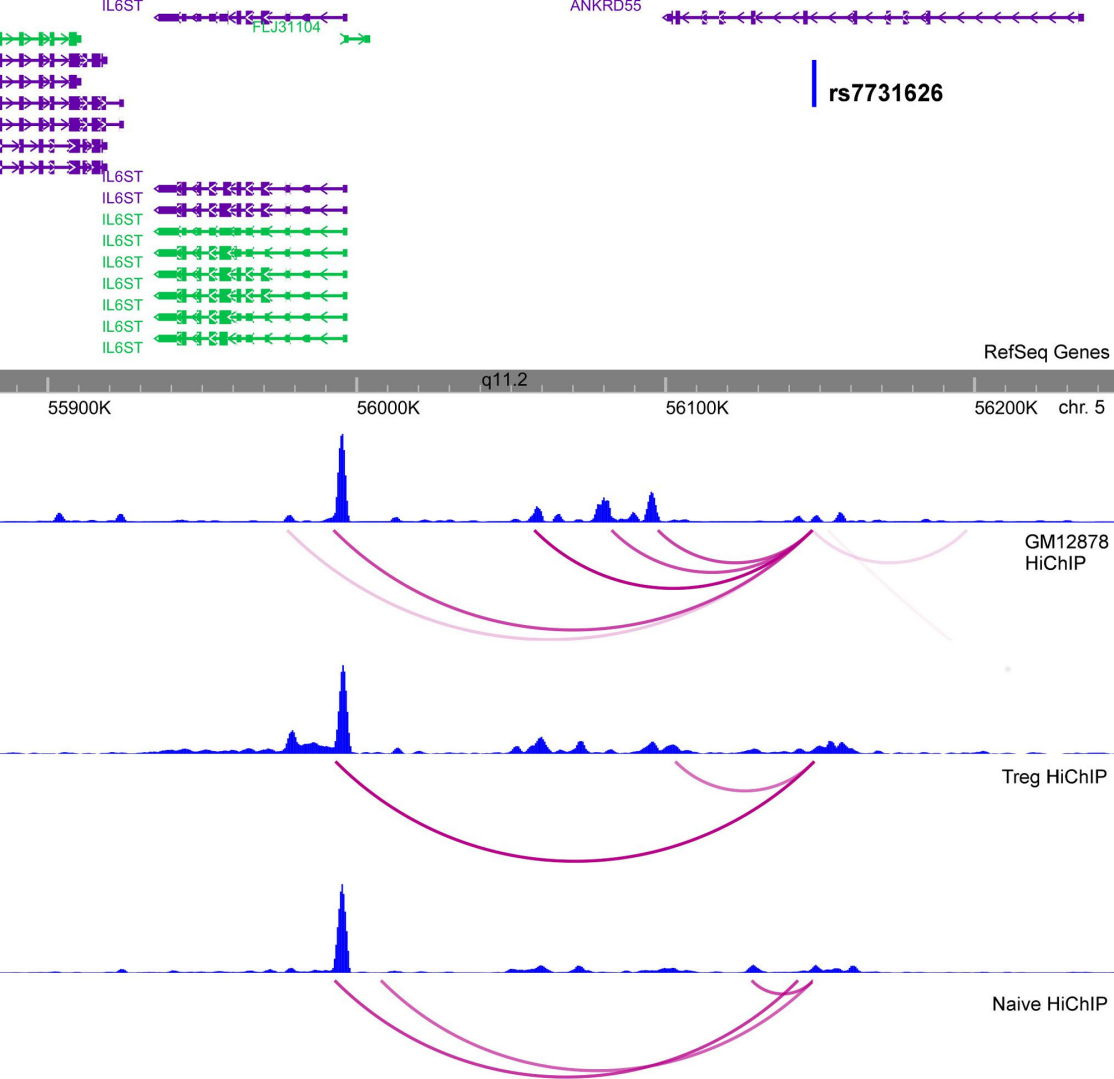
Chromatin interaction analysis at the *ANKRD55* locus. H3K27ac HiChIP signal at 5q11.2 showing enhancer-promoter chromatin interaction of rs7731626 to *IL6ST*. Blue graphs represent overlap with H3K27ac ChIP-seq peaks.

Interestingly, our analysis allowed us to refine the target gene of the association signal at 5q11.2 to *IL6ST*, since the credible SNP (rs7731626) showed chromatin contacts to the promoter of this gene but we did not observe interactions to the classically reported gene *ANKRD55* (figure 4). This exemplifies the potential of integrative analyses in deciphering the plausible mechanistic effect of association signals.

In total, we found 11 JIA targets genes showing both significant eQTL and H3K27ac HiChIP evidence.

## Discussion

We used a Bayesian model selection approach to demonstrate extensive sharing of JIA susceptibility loci across the ILAR subtypes and subsequent joint analysis of subtypes led to the identification of five novel risk loci, bringing the total of genome-wide significant regions for JIA to 22. We were able to prioritise causal genes at six loci integrating Bayesian fine-mapped credible SNPs, transcriptomics and chromatin interaction maps derived from disease-relevant cells.

A key challenge for studies investigating JIA susceptibility is how to account for the clinical heterogeneity across the ILAR clinical subtypes. Previous studies have focussed on the more frequent ILAR subtypes in an attempt to mitigate the loss of power due a non-specific phenotype definition.^26^ However, in the present study this would have resulted in the exclusion of 30% of the available cases. Guided by the Bayesian model selection, we chose to perform a combined analysis across all ILAR subtypes, which we show maximises power to detect novel loci. However, it is important to recognise that this approach will only increase power to detect loci that underlie biological pathways shared by multiple ILAR subtypes and does not exclude the existence of subtype specific risk factors, which are known to exist.^7 9 27^

Enrichment of JIA susceptibility loci in functional annotations highlighted that most association signals affect disease risk through regulatory effects on gene expression and in a cell-type specific manner. Our analysis pointed to the TFBS of RELA and EBF1 as two main non-redundant regulatory elements suggesting a crucial contribution of them in JIA risk. Interestingly, RELA and EBF1 are known to regulate Treg-induced tolerance^28^ and B cell specification and commitment,^29^ respectively.

Identifying target genes of the association signals is a crucial step to translate statistical findings to biological meaning and, in turn, for the development of new therapeutic strategies. Applying an integrative approach, we provide robust evidence to nominate target genes at the novel locus at 16p11.2. This is a known susceptibility locus for multiple chronic inflammatory diseases and includes attractive biological candidate genes such as *IL27*. However, complementary evidence from eQTL and chromatin data implicates the genes *CLN3* and *SULT1A2. CLN3* encodes a protein that is involved in lysosomal function suggesting a role for lysosome-mediated degradative pathways via autophagy and phagocytosis. Interestingly, Peeters *et al* reported that synovial fluid T cells derived from JIA patients showed enhanced autophagy.^30^ *SULT1A2* encodes a catalytic enzyme that sulfonates different molecular components like thyroid hormones. Therefore, this target gene may establish a link for the comorbidity observed between rheumatic conditions and thyroid disorders. A second example of successful refinement is the association signal at 5q11.2 to *IL6ST*, instead of the classically reported gene *ANKRD55*.^6^ We found that the *ANKRD55* intronic SNP, rs7731626, interacts with the promoter of *IL6ST*, and that its risk allele increases the expression of the gene. IL6ST is the interleukin 6 (IL-6) signal transducer and is the drug target of satralizumab, a biological drug that is currently in Phase III of a clinical trial for neuromyelitis optica, a rare autoimmune disease of the nervous system.^31^ Considering that other biological drugs targeting the IL-6 pathway, such as tocilizumab, are currently in use for the treatment of JIA, our findings provide genetic support for the study of satralizumab as a new therapeutic target for JIA.

In conclusion, our results highlight the utility of joint analysis considering all JIA subtypes to maximise discovery, shifting the classical paradigm on which previous JIA genetic studies were based, and illustrate the potential of integrative approaches to gain further insights into the genetic susceptibility of the disease, which may in turn inform future therapeutic drug targets and pathways.
