# Supplementary material for: Combined genetic analysis of juvenile idiopathic arthritis clinical subtypes identifies novel risk loci, target genes and key regulatory mechanisms
Source: Ann Rheum Dis. 2020 Oct 26;80(3):321–8. doi: 10.1136/annrheumdis-2020-218481 (PMC7892389; doi:10.1136/annrheumdis-2020-218481)
Supplement: Supplementary data [file annrheumdis-2020-218481supp002.pdf]

# SUPPLEMENTARY MATERIAL

## TABLE OF CONTENTS

1.- Online Supplementary Methods ..…………………………….………….….…2

1.1 Study cohort ……………………………………………….……………2

1.2 Genotyping and quality control ………………………………………....2

1.3 Imputation ………………………………………………………………3

1.4 Association testing and meta-analysis …………………………………..4

1.5 Heritability estimation …………………………………………………..4

1.6 Clinical subtype specificity ……………………………………………..4

1.7 Statistical fine-mapping of JIA-associated loci ………………………...5

1.8 Functional annotation enrichment analysis …………………………….5

1.9 Gene prioritisation with eQTL …………………………………………6

1.10 Chromatin interaction analysis in human B and T cell types ………...6

2.- Supplementary Figure Legends …………………...…………………….………8

3.- Supplementary Figures ………………….............................................................9

4.- Supplementary References……………………………………………………13



López-Isac E, *et al. Ann Rheum Dis* 2021; 80:321–328. doi: 10.1136/annrheumdis-2020-218481

# 1. ONLINE SUPPLEMENTARY METHODS

## 1.1 Study cohort

A total of 4,520 UK juvenile idiopathic arthritis (JIA) samples were recruited from multiple sources: the British Society for Paediatric and Adolescent Rheumatology (BSPAR) National Repository of JIA, the Childhood Arthritis Prospective Study (CAPS), the Childhood Arthritis Response to Medication Study (CHARMS), the UK JIA Genetics Consortium (UKJIAGC), The Biologics for Children with Rheumatic Diseases (BCRD) study, and the British Society for Paediatric and Adolescent Rheumatology Etanercept Cohort Study (BSPAR-ETN), a group of UK cases with long-standing JIA as described previously[1]. JIA participants were recruited with ethical approval and provided informed consent, including from the North West Multi-centre for Research Ethics Committee (MREC:02/8/104 and MREC:99/8/84), West Midlands Multi-centre Research Ethics Committee (MREC:02/7/106), North West Research Ethics Committee (REC:09/H1008/137) and the NHS Research Ethics Committee (REC:05/Q0508/95). JIA cases were classified according to the International League of Associations for Rheumatology (ILAR) criteria[2] (Supplementary Table 1). Healthy controls data on 9,965 individuals was obtained from the UK Household Longitudinal Study (https://www.understandingsociety.ac.uk/) accessed via the European Genome-phenome Archive.

## 1.2 Genotyping and quality control

JIA DNA samples were genotyped on the Illumina Infinium CoreExome and Infinium OnmiExpress genotyping arrays in accordance to the manufacturer's instructions. Genotype calling was performed by the GenCall algorithm in the GenomeStudio Data Analysis software platform (Genotyping Module v1.8.4). Preliminary genotype clustering was performed using the default Illumina cluster file to identify poor quality samples (call rate < 0.90). Following exclusion of low-quality samples, automated reclustering was performed to calibrate genotype

clusters based on the study samples. Sample-level quality control (QC) was performed based on the following exclusion criteria: final call rate < 0.98, outlier based on autosomal heterozygosity (2 standard deviations from the mean) and discrepancy between genetically inferred sex and database records. Single-nucleotide polymorphisms (SNPs) were excluded if they were non-autosomal, had a call rate < 0.98 or a minor allele frequency (MAF) < 0.01. Healthy controls were genotyped at the Wellcome Trust Sanger Institute using the Illumina Infinium CoreExome genotyping array. Sample and SNP QC was consistent with that described above for JIA case samples.

The two datasets were combined retaining the intersection of SNPs. Identity-by-descent was used to identify related individuals (kinship coefficient > 0.0884) across all study samples performed with the KING software package (version 1.9)[3]. For each related pair, the sample with the highest call rate was preferentially retained. Individuals were excluded if they were identified as outliers based on ancestry using principal component analysis (PCA) performed with the flashpca software package (version 2.0) where outliers were identified using aberrant R library (version 1.0)[4,5].

The total number of individuals that remained in the final QC-filtered dataset was 12,501 (3,305 cases and 9,196 healthy controls).

## 1.3 Imputation

QC-filtered GWAS dataset was subjected to whole-genome genotype imputation. Prior to imputation, SNPs with ambiguous alleles (C/G and A/T) were excluded and remaining SNPs were aligned to the Haplotype Reference Consortium (HRC) panel (version 1.1) using the HRC imputation preparation tool (https://www.well.ox.ac.uk/~wrayner/tools/)[6]. Phasing and imputation were performed in the Michigan Imputation server using SHAPEIT2[7] and Minimac3[8] respectively, and HRC

panel for reference. Following imputation, SNPs were excluded based on a MAF < 0.01 and imputation quality ($r^2$) < 0.4.

**1.4 Association testing and meta-analysis**

Case-control association testing was performed by SNPTEST software package (version 2.5.2) using the score method to account for imputation uncertainty. Three principal components, calculated as described above following exclusion of outliers, were included as covariates to account for any residual population sub-structure. Lambda genomic control ($\lambda_{GC}$), corrected for sample size ($\lambda_{1000}$), was calculated to test for inflation of test statistics attributable to population stratification not accounted for in the analysis. Any SNP with a p-value < $5 \times 10^{-6}$ was selected for validation in GWAS summary statistics from an independent dataset of 2,751 JIA cases (oligoarticular arthritis (oligoJIA) and rheumatoid factor (RF)-negative polyarthritis (RF–polyJIA)) and 15,886 controls of European Ancestry[9]. An inverse variance weighted fixed effects meta-analysis was performed using the software package GWAMA (version 2.2.2)[10]. The presence of heterogeneity of odds ratios (ORs) across datasets was evaluated with the test statistics $I^2$ and Q.

**1.5 Heritability estimation**

SNP-based heritability estimates were calculated using GCTA based on imputed data for all JIA cases[11]. Imputed SNPs were stratified into linkage disequilibrium (LD) score bins and a genetic relationship matrix (GRM) was calculated separately for each bin followed by restricted maximum likelihood (REML) analysis performed on the multiple GRMs.

**1.6 Clinical subtype specificity**

The specificity and sharing of JIA susceptibility SNPs across ILAR subtypes was interrogated using Bayesian multinomial logistic regression assuming an additive model implemented in the software package Trinculo (version 0.96) using default correlation priors (0.04) for all index SNPs with p-value < $5 \times 10^{-6}$[12]. In addition, we have included previously

reported JIA susceptibility SNPs based on analysis of a combined oligoJIA and RF-polyJIA cohort[13]. Trinculo identifies the best disease-specific model, the best sharing model, and estimates the Bayes factor between them at each analysed locus. Model selection was based on comparison of log-Bayes factors where a positive log-Bayes factor for specificity was interpreted as evidence that a particular association is specific to an ILAR subtype and a negative value indicative of sharing across multiple ILAR subtypes. The undifferentiated subtype was not included in this analysis.

**1.7 Statistical fine-mapping of JIA-associated loci**

The boundaries of independently associated loci were defined by a genetic distance of 0.1 centimorgans (cM) upstream and downstream of each lead SNP using HapMap fine-scale recombination rate estimates. Statistical fine-mapping of the association signal within each locus was performed using the FINEMAP software package (version 1.3.1) using the shotgun stochastic search function to identify independent effects using an LD panel of 4,000 randomly selected controls from the Understanding Society dataset[14]. The 95% credible SNP sets for each locus were selected on the summation of the posterior inclusion probabilities (PIPs) for the most likely causal SNPs. Any independent effects within each locus identified by the stochastic search approach were verified by conditional and joint analysis using GCTA-COJO with the same LD panel as FINEMAP (version 1.92.4)[15]. All identified credible SNPs were annotated with gene location based on RefSeq transcript and evidence for association with other diseases using data from the GWAS catalogue[16]. Non-synonymous credible SNPs were annotated with the pre-calculated Combined Annotation Dependent Depletion (CADD) raw score and scaled score from dbNSFP (version 3.3a)[17]. Annotation was performed with ANNOVAR (version 2019Oct24)[18].

**1.8 Functional annotation enrichment analysis**

Summary statistics from the association analysis of all ILAR subtypes were tested for enrichment in four categories of annotations based on experimental functional genomic data including gene structure (coding sequence (CDS), 3`UTR and 5`UTR) from the GENCODE Project, binding sites for 165 transcription factors from the ENCODE Project, and enhancers and active promoters for 98 cell types derived from the Roadmap Epigenomics Project[19–21]. Enrichment of JIA associations were tested separately in each annotation using fgwas (version 0.3.6)[22]. A joint model of independent enrichments was identified using a forward selection approach of selecting the most significant annotation and sequentially adding additional significant annotations and retaining those that significantly increase the likelihood of the joint model using cross-validation with penalised likelihood to identify the model with the highest cross-validation likelihood.

### 1.9 Gene prioritisation with eQTL

Expression quantitative trait locus (eQTL) data for 15 cell types was downloaded from the DICE (Database of Immune Cell Expression, eQTLs and Epigenomics) project website[23]. Cell types consisted of three innate immune cell types ($CD14^{high}$ $CD16^{-}$ classical monocytes, $CD14^{-}$ $CD16^{+}$ non-classical monocytes, $CD56^{dim}$ $CD16^{+}$ natural killer (NK) cells), four adaptive immune cell types that have not encountered cognate antigen in the periphery (naive B cells, naive $CD4^{+}$ T cells, naive $CD8^{+}$ T cells, and naive regulatory T cells [Treg]), six $CD4^{+}$ memory or more differentiated T cell subsets ($T_H1$, $T_H1/17$, $T_H17$, $T_H2$, follicular helper T cell [TfH], and memory Treg [mTreg]), and two activated cell types (naive $CD4^{+}$ and $CD8^{+}$ T cells that were stimulated *ex vivo*) (description from DICE website https://dice-database.org/). Correlation of susceptibility association signals and gene expression were identified by selecting the top eQTL SNP for each gene in each eQTL dataset and retaining those that were also present in the combined list of all FINEMAP credible SNPs for



López-Isac E, *et al. Ann Rheum Dis* 2021; 80:321–328. doi: 10.1136/annrheumdis-2020-218481

associated loci. Colocalisation of the susceptibility association and eQTL signals was then confirmed using the coloc R package using approximate Bayes factors[24].

**1.10 Chromatin interaction analysis in human B and T cell types**

Prioritisation of causal genes was further complemented by the interrogation of chromatin interaction data for SNPs correlated with eQTL signals. H3K27ac HiChIP data in GM12878, primary human naïve T cells ($CD4^+CD45RA^+CD25^-CD127^{high}$), regulatory T (Treg) cells ($CD4^+CD25^+CD127^{low}$) and $T_H17$ cells ($CD4^+CD45RA^-CD25^-CD127^{high}CCR6^+CXCR5^-$) were interrogated to identify target genes of JIA-associated regions[25]. In detail, sequencing data for the HiChIP libraries was filtered and the adapters were removed using fastp v0.19.4[26]. Then we mapped the reads to the human reference genome GRCh38 with Hi-C Pro v2.11.0 using default settings[27]. HiChIP-peaks v0.1.1 was used with default settings and false discovery rate (FDR) $< 0.01$ to identify H3K27ac peaks enriched regions[28]. Identification of significant chromatin loops was performed with FitHiChIP using the following settings: Coverage normalization, stringent background with merging enabled, peaks generated from HiChIP-peaks and 5kb bin size[29]. We explored chromatin interaction profiles of the credible SNPs sets from each locus. To identify the connectivity of credible SNPs to target genes, we subsetted the interactions to those linking a transcription start site (TSS) and SNPs overlapping a H3K27ac HiChIP peak in a 5kb resolution. We also nominated the genes for which the TSS was within 1kb of a credible SNP overlapping an H3K27ac peak as identified from HiChIP data. In addition, we explored chromatin interaction maps obtained by capture Hi-C experiments in GM12878 and Jurkat cell types[30]. Credible SNPs were set as anchor points to identify physical interactions between restriction fragments containing the variants and gene promoters using IRanges and GenomicRanges R packages.



López-Isac E, *et al. Ann Rheum Dis* 2021; 80:321–328. doi: 10.1136/annrheumdis-2020-218481

## 2. SUPPLEMENTARY FIGURE LEGENDS

**Supplementary Figure 1. Bayesian model selection analysis for ILAR subtype specificity of JIA susceptibility SNPs.** Comparison of log Bayes factor for best specific ILAR subtype model (x axis) and best shared model (y axis). Susceptibility SNPs (represented by blue dots for loci from this study, red dots for previously published SNPs based on oligoJIA and RF-polyJIA) above the grey line indicate evidence for sharing of association across multiple ILAR subtypes.

**Supplementary Figure 2. Enrichment of JIA susceptibility SNPs in transcription factor binding sites.** Annotations showing significant enrichment are displayed in green.

**Supplementary Figure 3. Enrichment of JIA susceptibility SNPs in regulatory regions of 98 cells.** Supplementary Table 5 provides the correspondence between cell codes and cell types.

**Supplementary Figure 4. Enrichment of JIA susceptibility SNPs in active promoters of 98 cells.** Supplementary Table 6 provides the correspondence between cell codes and cell types.



López-Isac E, *et al. Ann Rheum Dis* 2021; 80:321–328. doi: 10.1136/annrheumdis-2020-218481

## 3. SUPPLEMENTARY FIGURES

### Supplementary Figure 1

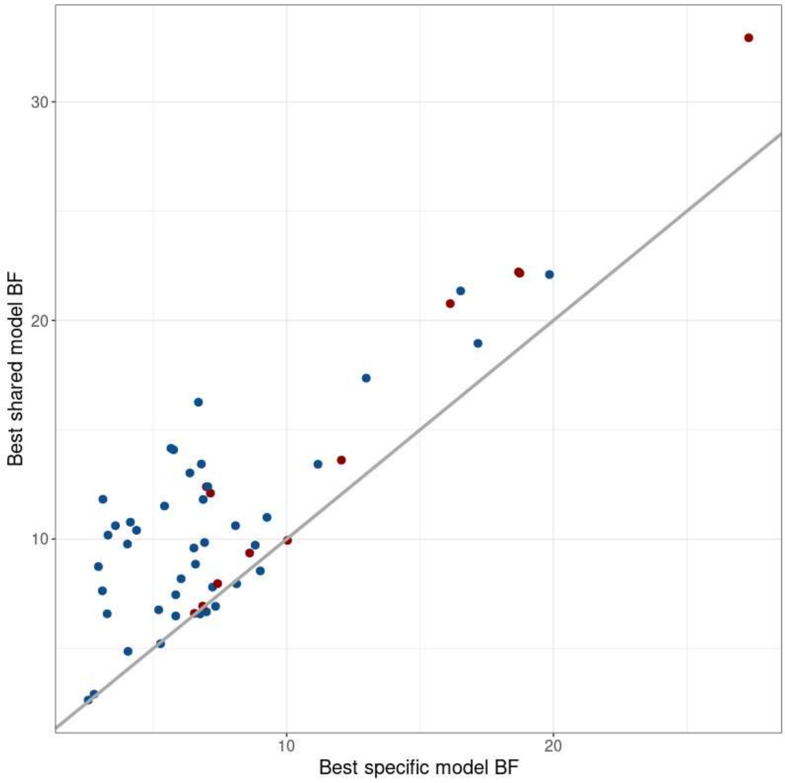

**Supplementary Figure 2**

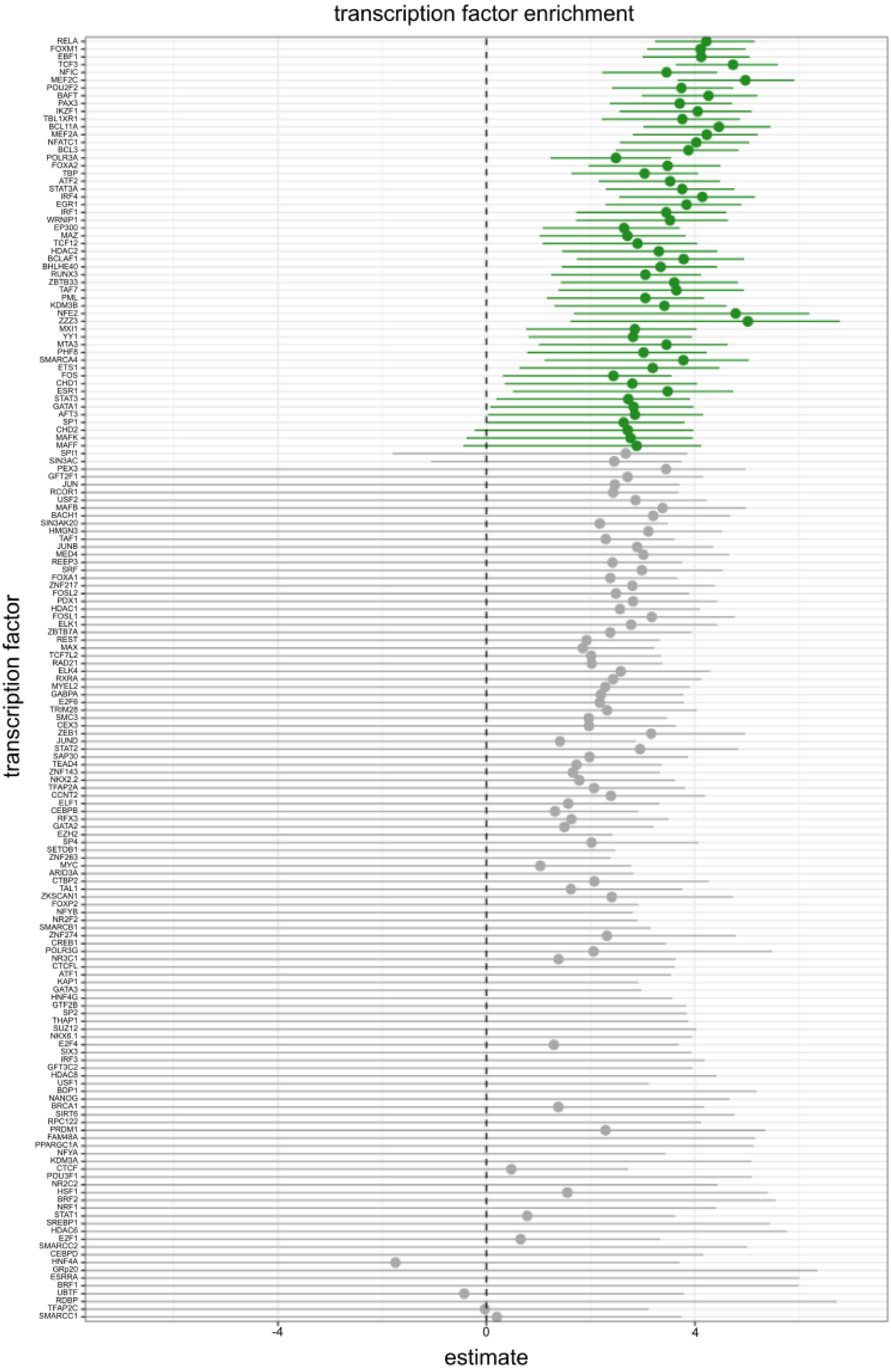




López-Isac E, *et al. Ann Rheum Dis* 2021; 80:321–328. doi: 10.1136/annrheumdis-2020-218481

**Supplementary Figure 3**

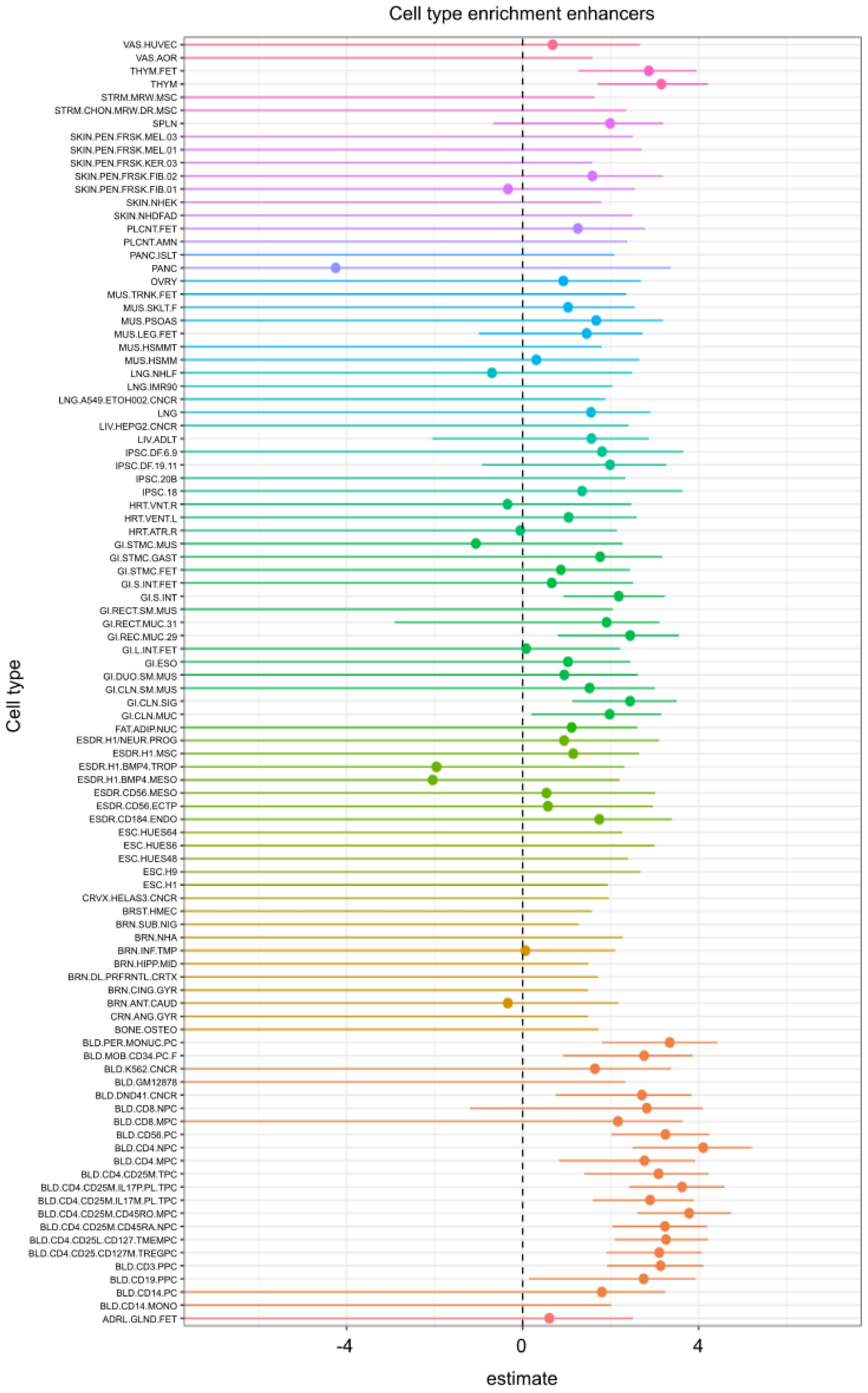

## Supplementary Figure 4

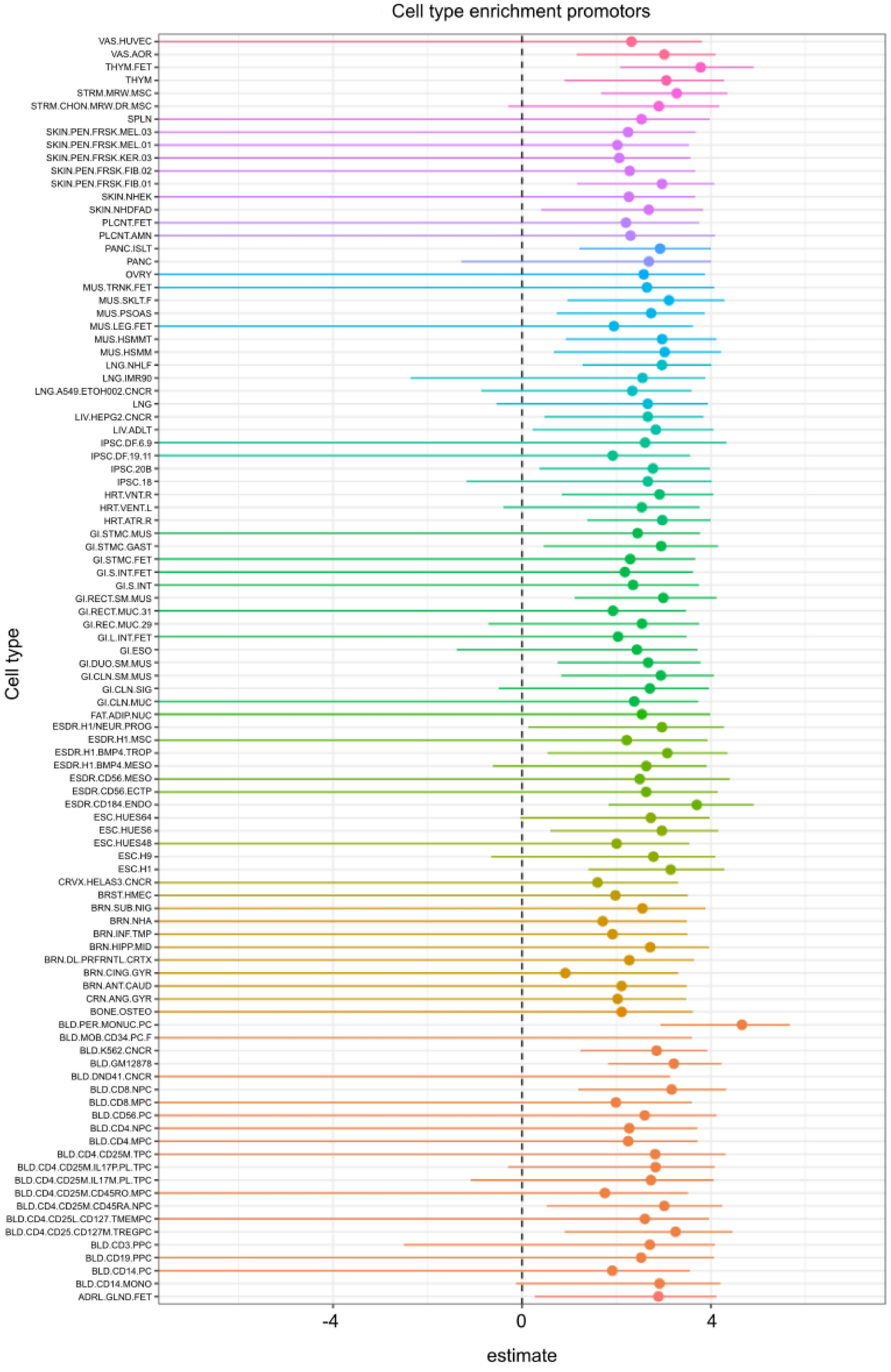




López-Isac E, *et al. Ann Rheum Dis* 2021; 80:321–328. doi: 10.1136/annrheumdis-2020-218481

## 4. SUPPLEMENTARY REFERENCES


1 Packham JC, Hall MA. Long-term follow-up of 246 adults with juvenile idiopathic arthritis: Functional outcome. *Rheumatology* 2002;**41**:1428–35. doi:10.1093/rheumatology/41.12.1428

2 Petty RE, Southwood TR, Manners P, *et al.* International League of Associations for Rheumatology classification of juvenile idiopathic arthritis: second revision, Edmonton, 2001. *J Rheumatol* 2004;**31**:390–2.

3 Manichaikul A, Mychaleckyj JC, Rich SS, *et al.* Robust relationship inference in genome-wide association studies. *Bioinformatics* 2010;**26**:2867–73. doi:10.1093/bioinformatics/btq559

4 Abraham G, Qiu Y, Inouye M. FlashPCA2: principal component analysis of Biobank-scale genotype datasets. *Bioinformatics* 2017;**33**:2776–8. doi:10.1093/bioinformatics/btx299

5 Bellenguez C, Strange A, Freeman C, *et al.* A robust clustering algorithm for identifying problematic samples in genome-wide association studies. *Bioinformatics* 2012;**28**:134–5. doi:10.1093/bioinformatics/btr599

6 McCarthy S, Das S, Kretzschmar W, *et al.* A reference panel of 64,976 haplotypes for genotype imputation. *Nat Genet* 2016;**48**:1279–83. doi:10.1038/ng.3643

7 Delaneau O, Zagury JF, Marchini J. Improved whole-chromosome phasing for disease and population genetic studies. *Nat Methods* 2013;**10**:5–6. doi:10.1038/nmeth.2307

8 Das S, Forer L, Schönherr S, *et al.* Next-generation genotype imputation service and methods. *Nat Genet* 2016;**48**:1284–1287. doi:10.1038/ng.3656

9 McIntosh LA, Marion MC, Sudman M, *et al.* Genome-Wide Association Meta-Analysis Reveals Novel Juvenile Idiopathic Arthritis Susceptibility Loci. *Arthritis Rheumatol (Hoboken, NJ)* 2017;**69**:2222–32. doi:10.1002/art.40216

10 Magi R, Morris AP. GWAMA: software for genome-wide association meta-analysis. *BMC Bioinformatics* 2010;**11**:288. doi:10.1186/1471-2105-11-288

11 Yang J, Manolio TA, Pasquale LR, *et al.* Genome partitioning of genetic variation for complex traits using common SNPs. *Nat Genet* 2011;**43**:519–25. doi:10.1038/ng.823

12 Jostins L, McVean G. Trinculo: Bayesian and frequentist multinomial logistic regression for genome-wide association studies of multi-category phenotypes. *Bioinformatics* 2016;**32**:1898–900. doi:10.1093/bioinformatics/btw075

13 Hinks A, Cobb J, Marion MC, *et al.* Dense genotyping of immune-related disease

regions identifies 14 new susceptibility loci for juvenile idiopathic arthritis. *Nat Genet* 2013;**45**:664–9. doi:10.1038/ng.2614

14 Benner C, Spencer C, Havulinna AS, *et al.* FINEMAP: efficient variable selection using summary data from genome-wide association studies. *Bioinformatics* 2016;**32**:1493–501. doi:10.1093/bioinformatics/btw018

15 Yang J, Ferreira T, Morris AP, *et al.* Conditional and joint multiple-SNP analysis of GWAS summary statistics identifies additional variants influencing complex traits. *Nat Genet* 2012;**44**:369–75, S1-3. doi:10.1038/ng.2213

16 MacArthur J, Bowler E, Cerezo M, *et al.* The new NHGRI-EBI Catalog of published genome-wide association studies (GWAS Catalog). *Nucleic Acids Res* 2017;**45**:D896–D901. doi:10.1093/nar/gkw1133

17 Liu X, Wu C, Li C, *et al.* dbNSFP v3.0: A One-Stop Database of Functional Predictions and Annotations for Human Nonsynonymous and Splice-Site SNVs. *Hum Mutat* 2016;**37**:235–41. doi:10.1002/humu.22932

18 Yang H, Wang K. Genomic variant annotation and prioritization with ANNOVAR and wANNOVAR. *Nat Protoc* 2015;**10**:1556–66. doi:10.1038/nprot.2015.105

19 Harrow J, Frankish A, Gonzalez JM, *et al.* GENCODE: the reference human genome annotation for The ENCODE Project. *Genome Res* 2012;**22**:1760–74. doi:10.1101/gr.135350.111

20 ENCODE Project Consortium I, Kundaje A, Aldred SF, *et al.* An integrated encyclopedia of DNA elements in the human genome. *Nature* 2012;**489**:57–74. doi:10.1038/nature11247

21 Chadwick LH. The NIH Roadmap Epigenomics Program data resource. *Epigenomics* 2012;**4**:317–24. doi:10.2217/epi.12.18

22 Pickrell JK. Joint analysis of functional genomic data and genome-wide association studies of 18 human traits. *Am J Hum Genet* 2014;**94**:559–73. doi:10.1016/j.ajhg.2014.03.004

23 Schmiedel BJ, Singh D, Madrigal A, *et al.* Impact of Genetic Polymorphisms on Human Immune Cell Gene Expression. *Cell* 2018;**175**:1701-1715.e16. doi:10.1016/j.cell.2018.10.022

24 Giambartolomei C, Vukcevic D, Schadt EE, *et al.* Bayesian Test for Colocalisation between Pairs of Genetic Association Studies Using Summary Statistics. *PLoS Genet* 2014;**10**:e1004383. doi:10.1371/journal.pgen.1004383

25 Mumbach MR, Satpathy AT, Boyle EA, *et al.* Enhancer connectome in primary human

cells identifies target genes of disease-associated DNA elements. *Nat Genet* 2017;**49**:1602–12. doi:10.1038/ng.3963

26 Chen S, Zhou Y, Chen Y, *et al.* fastp: an ultra-fast all-in-one FASTQ preprocessor. *Bioinformatics* 2018;**34**:i884–90. doi:10.1093/bioinformatics/bty560

27 Servant N, Varoquaux N, Lajoie BR, *et al.* HiC-Pro: an optimized and flexible pipeline for Hi-C data processing. *Genome Biol* 2015;**16**:259. doi:10.1186/s13059-015-0831-x

28 Shi C, Rattray M, Orozco G. HiChIP-Peaks: A HiChIP peak calling algorithm. *bioRxiv* Published Online First: 2019. doi:10.1101/682781

29 Bhattacharyya S, Chandra V, Vijayanand P, *et al.* Identification of significant chromatin contacts from HiChIP data by FitHiChIP. *Nat Commun* 2019;**10**:4221. doi:10.1038/s41467-019-11950-y

30 Martin P, McGovern A, Orozco G, *et al.* Capture Hi-C reveals novel candidate genes and complex long-range interactions with related autoimmune risk loci. *Nat Commun* 2015;**6**:10069. doi:10.1038/ncomms10069
